# Insights into the Intrinsic Factors Affecting the NIR Reflectance Based on Rylene Diimide Molecules

**DOI:** 10.3390/ma14185269

**Published:** 2021-09-13

**Authors:** Weili Zeng, Yujie Song, Jianning Zhang, Hong Chen, Ming Liu, Wangqiao Chen

**Affiliations:** 1 Guangdong Provincial Key Laboratory of Optical Information Materials and Technology, National Center for International Research on Green Optoelectronics, South China Academy of Advanced Optoelectronics, South China Normal University, Guangzhou 510006, China; 2019023280@m.scnu.edu.cn (W.Z.); 2020023689@m.scnu.edu.cn (H.C.); 2 Engineering Laboratory of Advanced Energy Materials, Ningbo Institute of Materials Technology & Engineering, Chinese Academy of Sciences, Ningbo 315201, China; songyujie@nimte.ac.cn (Y.S.); zhangjianning@nimte.ac.cn (J.Z.); 3 School of Chemistry and Chemical Engineering, Harbin Institute of Technology, Harbin 150001, China

**Keywords:** NIR reflectance, rylene diimide, conjugated backbone, crystallinity

## Abstract

A clear understanding of the relationships between molecular structure and NIR reflectance (700–2500 nm) behavior is important and highly desirable for developing appropriate NIR-reflective materials to combat NIR heat radiation from sunlight. In this research, three groups of imide-based compounds have been adopted to investigate the influence of the intrinsic molecular structures on the NIR-reflective properties. It is found out that for the compounds with alkyl groups, the NIR reflectance will increase as the degree of the conjugated backbone increases, especially for the reflectance from 1750 nm to 2500 nm. In addition, despite that the alkyl or amine groups deteriorate the NIR reflectance, the NIR reflectance varies within a certain interval and the isomers with branched alkyl groups show identical or smaller NIR reflectance than those of isomers with linear alkyl groups. For different compounds, crystallinity seems to almost have no relationship with their NIR reflectance.

## 1. Introduction

Near-infrared (NIR) radiation (700–2500 nm) accounts for 52% of the solar energy from sunlight, and significantly contributes to the energy accumulation and temperature elevation of various man-made concrete buildings, which not only results in the urban heat island (UHI) effect, but also greatly increases the electricity energy consumption (i.e., cooling) [1,2,3,4]. Thus, it is highly desirable to address this issue through developing effective NIR reflective coating materials.

**Figure 1:**
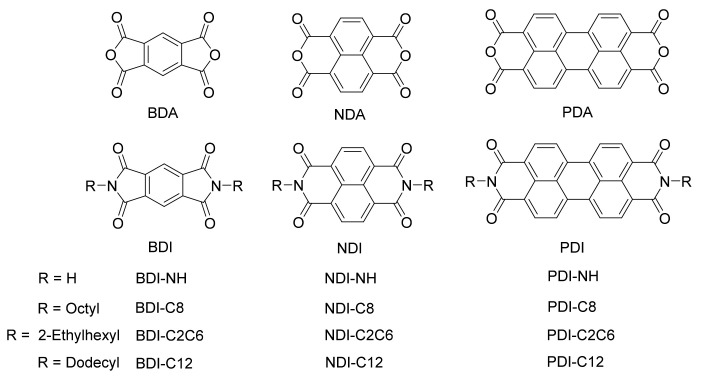
Chemical structures of the aromatic diimide-based compounds studied in this research.

Generally, NIR reflective pigments can be classified into two groups: inorganic compounds [5,6,7,8] (i.e., titanium oxide, chromium oxide, and rare-earth oxide, etc.,) and organic compounds [9,10,11,12] (i.e., perylenetetracarboxylic diimide (PDI), azo-compounds, and copper phthalocyanine). Compared to certain inorganic counterparts, organic pigments are becoming the research focus due to their low toxicity, biocompatibility, and cost effectiveness [13,14,15,16,17,18,19]. Especially for some of the aromatic pigments, such as perylene pigments, they are essentially nontoxic, with little or no threat to human health or the environment, and are not considered in the US to be hazardous under OSHA’s Hazard Communication Standard [13]. Moreover, organic reflective materials can be readily solubilized in common organic solvents, which endows them to have better processibility and miscibility with polymer matrix [20,21,22]. Actually, for certain organic compounds with suitable molecular structure, such as thiophene-fused-heteroaromatic diones, they exhibited relatively good stability and almost had no decomposition after exposing to air for three months [20]. Among the available organic pigments for cooling, PDI derivatives represent the most investigated coating components due to their high thermal stability, tunable optical and energy levels, good solubilities and dispersion properties [23,24]. The factors that influence NIR reflectance behaviors of PDI derivatives include particle size, N-substituted functional groups, crystallinity, and aggregation state, which have been extensively studied [25,26,27,28,29,30,31,32]. For example, Kaur et al. synthesized five PDI-based pigments and investigated the correlation between NIR reflectance and several parameters (i.e., particle size, crystallinity, dipole moment etc.,). However, they did not correlate the nature of the N-substituents with NIR reflectance due to the limited number of the investigated pigments [25]. Martini et al. compared the NIR reflectance of another PDI derivative (Paliogen^®^ Black L0086, in direct commercially-available form and the as-synthesized form from lab). The authors found that compared with the commercial form, the NIR reflectance could be doubled (from 25% to 50%) by increasing the crystallinity and structure order through lab synthesis [31]. However, in several other literatures, the researchers believed that no correlation between the NIR properties and the crystallinity of different PDI-derived pigments could be established [28,29]. Because most former research mainly focused on the PDI-based NIR reflective materials and, due to the limited studied molecular systems, the relationship between molecular structures and NIR-reflective properties has not been fully understood and the key factors influencing the NIR reflectance is still unclear. Moreover, in the previous studies, the sample films were mostly prepared by taking a certain polymer as the matrix or carrier, which would inevitably affect the NIR reflectance of the sample in the pure form due to the ubiquitously existential Van der Waals force. Such preparation may partially contribute to the contradicting conclusion reported in the former studies. Such gap strongly encourage us to expand the scope of the studied molecules through synthesizing three groups of dyes based on 1,2,4,5-benzenetetracarboxylic 1,2,4,5-diimide (BDI), naphthalene-1,4,5,8-tetracarboxylic diimide (NDI), and PDI backbones as shown in Figure 1, and study their NIR reflective properties thoroughly. Moreover, we also intend to test the NIR reflectance of the as-prepared samples in the pure form without any external additives or components [33]. Such measurement would make it easier for us to compare the intrinsic NIR reflective behaviors of these molecules according to their gradually increased conjugated backbones from BDI to NDI, then to PDI.

## 2. Results and Discussion

The anhydride compounds (1,2,4,5-benzenetetracarboxylic 1,2,4,5-dianhydride (BDA), naphthalene-1,4,5,8-tetracarboxylic dianhydride (NDA) and perylenetetracarboxylic dianhydride (PDA)) are commercially available and used as received. The diimide derivatives were synthesized according to the reported literatures [25,26,34,35,36,37]. All as-prepared materials were confirmed through ^1^ H NMR spectra as shown in Figure S1.

**Figure 2:**
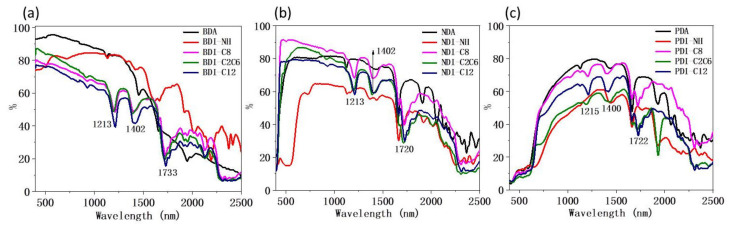
NIR reflectance spectra for three groups of: (**a**) BDI-, (**b**) NDI-, and (**c**) PDI-based compounds.

The reference NIR reflectance was measured from 400 nm to 2500 nm as shown in Figure S1. NIR reflectance of three groups of BDI-, NDI-, and PDI-based compounds were measured to investigate the influence of the intrinsic molecular structure on their NIR reflectance behavior on a UV-Vis-NIR Lambda 950 instrument [20,33] (Figure 2). The wavelength from 700 nm to 1750 nm accounts for higher than 80% of the NIR solar energy for heat production [1]. Hence, the whole NIR reflectance spectrum was divided into two parts: 700–1750 nm and 1750–2500 nm and the average NIR reflectance of 700–1750 nm and 1750–2500 nm for BDI-, NDI-, and PDI-based compounds are summarized in Table 1.

As shown in Figure 2, for all three groups of BDI-, NDI-, and PDI-based molecules, when the main conjugated backbone is fixed, the NIR reflectance is almost independent on the N-functional groups. The NIR reflectance variation is just confined to a certain interval. In other words, when we compare the largest and smallest intensity of the NIR reflectance for any two molecules with the same backbone at any wavelength from 700 to 2500 nm, the variation of their NIR reflectance values is generally within 25%. For the detailed data, as shown in Table 1, the average reflectance of 700–1750 nm for all BDI, NDI, and PDI derivatives varies within 23% (77% for BDI-NH, 54% for BDI-C12), 19% (77% for NDI-C8, 58% for NDI-NH), and 21% (70% for PDA, 49% for PDI-NH) respectively and the same phenomenon also happens for the spectrum region from 1750 nm to 2500 nm, which might hint that for a particular compound, its NIR reflectance could be mainly determined by its conjugated backbone, while the change of its peripheral functional groups only lead to the variation of the reflectance within a limited range. This does not agree with the observation from some other reports where a large difference of the NIR reflectance value was observed when just changing the functional groups [28]. Meanwhile, the main peaks in the NIR reflectance spectra for all the BDI, NDI, and PDI compounds (Figure 1) appeared at similar position around 1210 nm, 1400 nm, and 1720 nm and the possible reason is explained in the following FTIR section.

Although some common features were observed in the NIR reflectance spectra for these three groups, their NIR reflectance did have difference in many aspects. First, as the UV-vis absorption red-shifted gradually from BDI to NDI and PDI due to their increased conjugation, their onset NIR reflectance were also redshifted gradually from below 400 nm for BDI to around 450 nm for NDI and higher than 600 nm for PDI. The reason is due to the gradually redshifted λ_onset_ of UV-vis absorption in this region, which is in agreement with the reported literatures [38,39]. Second, as shown in Table 1, NDI and PDI compounds containing C8, C2C6, and C12 alkyl groups generally exhibited higher average NIR reflectance than those of BDI compounds with the same groups through 700 nm to 2500 nm. Third, compared with NDI and PDI groups, the average NIR reflectance of BDI group decreased more sharply in the region from 1750 nm to 2500 nm and the average reflectance value is generally below 25%. Therefore, the second and third points discussed above indicate that for molecules with alkyl groups, as the degree of the conjugated backbone increased, it will contribute to the elevation of NIR reflectance simultaneously, especially for the NIR reflectance for the spectrum region from 1750 nm to 2500 nm.

**Figure 3:**
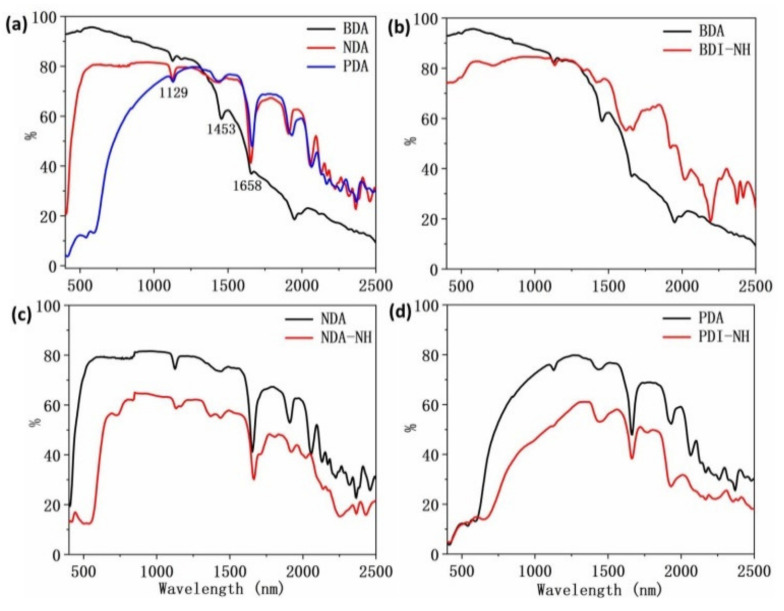
NIR reflectance spectra of: (**a**) BDA, NDA and PDA, (**b**) BDI-, (**c**) NDI-, and (**d**) PDI-based compounds with anhydride and amine group.

**Figure 4:**
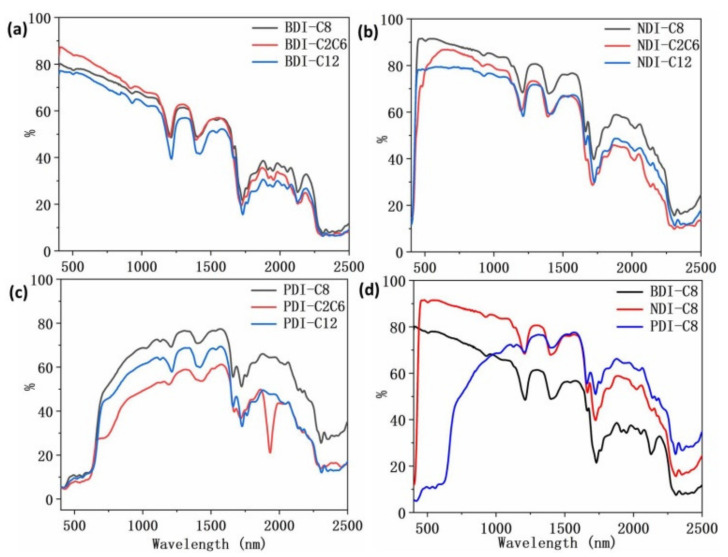
NIR reflectance spectra of: (**a**) BDI-, (**b**) NDI-, (**c**) PDI-based compounds with different alkyl groups, and (**d**) BDI, NDI and PDI with C8 alkyl group.

To further discuss the NIR reflectance of the three BDI, NDI, and PDI molecule groups in details, we divide these compounds into two groups: one with anhydride and NH (Figure 3) and the other with C8, C2C6, and C12 (Figure 4). As shown in Figure 3a, it is apparent that BDA showed much higher NIR reflectance than NDA and PDA between 400 nm and 1300 nm, with the highest NIR reflectance value up to 96% at 570 nm. A sharp drop in NIR reflectance of BDA (23% for 1750–2500 nm) was observed after 1300 nm and became significantly lower in the NIR reflectance intensity than NDA (45% for 1750–2500 nm) and PDA (45% for 1750–2500 nm). NDA, BDA, and PDA as well as BDA-NH, NDA-NH, and PDI-NH exhibited three NIR reflectance peaks at 1129 nm, 1453 nm, and 1658 nm with much smaller intensity different from Figure 2. As shown in Figure 3b–d, compared with BDA, NDA, and PDA, all the BDI-NH, NDI-NH, and PDI-NH exhibited redshift but lower NIR reflectance intensity except for BDI-NH, which showed an abrupt higher NIR reflectance at wavelength higher than 1600 nm and a high average reflectance value of 41% for 1750–2500 nm. The observations are in accordance with the theory proposed by Kaur et al. [27].

It was reported that isomers with the same molecular formula exhibited distinct NIR reflectance and longer alkyl groups may decrease the NIR reflectance [28]. Inspired by these studies, we investigated the NIR reflectance of BDI-, NDI-, and PDI-based compounds with different alkyl groups (Figure 4). As shown in Figure 4a–c and Table 1, all of BDI, NDI, and PDI derivatives with alkyl C8 group exhibited higher average NIR reflectance than BDI, NDI, and PDI derivatives with longer C12 group, irrespective of the spectrum region (700–1750 nm or 1750–2500 nm), indicating that for a particular series of compounds, longer alkyl groups may have adverse effect on the NIR reflectance intensity. The same trend was also observed for NDI-C8 and NDI-C2C6 as well as PDI-C8 and PDI-C2C6. The NIR reflectance of both NDI-C8 and PDI-C8 were higher than that of NDI-C2C6 and PDI-C2C6 throughout the NIR reflectance spectrum (from 700 nm to 2500 nm) (Figure 4b,c). Nevertheless, as shown in Figure 4a, NIR reflectance spectrum of BDI-C8 almost overlapped with that of BDI-C2C6 and both the NIR reflectance spectra showed nearly identical contour profile and similar average NIR reflectance (59% for BDI-C8 and 57% for BDI-C2C6) for 700–1750 nm. Based on the above observation, the isomers with branched alkyl groups showed identical or smaller NIR reflectance intensity than those of isomers with linear alkyl groups. As shown in Figure 4d, by the comparison of the NIR reflectance of BDI-C8, NDI-C8, and PDI-C8, one could clearly observe that BDI-C8 displayed smaller NIR reflectance intensity than those of NDI-C8 and PDI-C8 from 1000 nm to 2500 nm. From 1750 nm to 2500 nm, PDI-C8 showed apparently higher NIR reflectance than those of NDI-C8 and BDI-C8, which corresponded well with the higher average NIR reflectance of PDI-C8 (48%) from 1750 nm to 2500 nm.

There are many factors that affect the NIR reflectance of one dye, including the molecular structure, particle size, crystallinity etc. In the following section, we try to find the reasons that account for the different NIR reflectance behaviors for the three BDI-, NDI-, and PDI-based compounds. To study the relationship between one particular influencing factor and NIR reflectance, it is necessary to keep the other influencing factors constant. For example, in order to study the influence of molecular structure on the NIR reflectance, the other influencing factors, such as film morphology and particle size, must be kept the same. This is a challenging task because with the change of molecular structure, the other parameters such as film morphology and particle size may change simultaneously. Researchers tried to overcome this issue by dispersing the reflective pigment in a host polymer to produce uniform polymer film [25,29,31]. However, the introduction of an external matrix or carriers will inevitably bring uncertain factors into the hydride polymer due to the intermolecular interaction such as the Van der Waals force.

**Figure 5:**
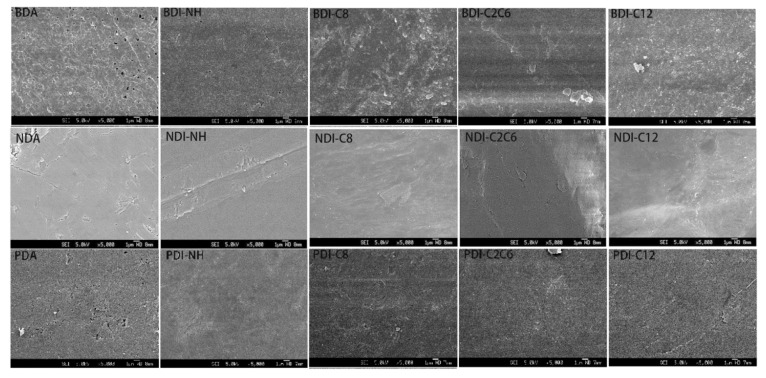
SEM pictures of the surfaces of as-prepared BDI, NDI, and PDI sheets.

**Figure 6:**
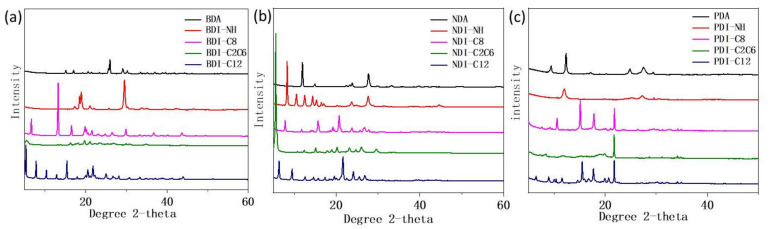
XRD spectra of: (**a**) BDI-, (**b**) NDI-, and (**c**) PDI-based compounds.

In order to get rid of the influence of different particle size as well as the surface morphology of the film on the NIR reflectance, the sample was ground with a mortar and pestle and pressed into thin pellet of similar size and thickness using the FTIR sample preparation method [33]. All the samples showed similar surface morphology under SEM observation due to the same iron sheet used to press the sample pellets (Figure 5). The XRD spectra of all BDI-, NDI-, and PDI-based compounds are measured as shown in Figure 6 to study their particle size situation and elucidate the effect of crystallinity on their NIR reflectance. The corresponding particle sizes and crystallinity data are summarized in Table 2. As shown in Table 2, most of the particle sizes are in the same order of magnitude ranging from 20 nm to 60 nm. Hence, the variation in the particle size is not sufficient to significantly affect the observed NIR reflectance and its influence on the variation of reflectance can be regarded as minimal in our research condition.

The crystallinity degrees of all compounds were calculated from their XRD patterns in agreement with the following equation [40,41]:

(1) $$Crystallinity\;degree=\frac{Ic}{Ic+Ia}$$

where *I_c_* is the area under the sharp peaks and attributed to the scattering intensity from the crystalline content, whereas *I_a_* is the area contributed from the related amorphous content. The calculated crystallinity degree values are summarized in Table 2.

As shown in Figure 3b–d and Table 1, the introduction of NH functional group into the molecules generally reduced the NIR reflectance. Assuming the crystallinity of the molecules plays a dominant role in the intensity of NIR reflectance, it can be speculated that the XRD spectra of BDA, NDA, and PDA should show higher crystallinity than their NH derivatives. However, the XRD signals did not agree with such speculation. As shown in Table 2, BDA (42%) and NDA (57%) showed lower crystallinity than BDI-NH (64%) and NDI-NH (71%) respectively, and only PDA (43%) shows slightly higher crystallinity than PDI-NH (40%). For BDI-, NDI-, and PDI-based compounds containing C8, C2C6, and C12 alkyl groups, C8-containing compounds all exhibited similar or higher NIR reflectance than its C2C6 and C12-containing counterparts as shown in Figure 4a–c and Table 1. Nevertheless, both BDI-C8 (60%) and NDI-C8 (61%) exhibited lower crystallinity than BDI-C2C6 (65%), BDI-C12 (70%) and NDI-C2C6 (71%), NDI-C12 (68%) respectively except PDI-C8 (64%) showed slightly higher crystallinity than PDI-C2C6 (53%) and PDI-C12 (62%). From the above discussion, substantial evidence proves that the crystallinity of the samples have no obvious direct relationship with the NIR reflectance when the studied compounds are based on the same conjugated backbone but with different functional groups.

**Figure 7:**
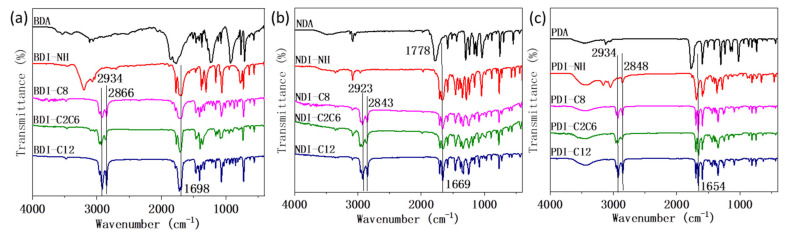
FTIR spectra of: (**a**) BDI-, (**b**) NDI-, and (**c**) PDI-based compounds.

In 2013 year, Kaur et al. found out that the overtones and combination modes resulted from high-frequency fundamental vibrations due to the alkyl groups and aromatic groups deteriorate the NIR reflectance [27]. This result encourages us to systematically investigate the FTIR of all the three groups of BDI-, NDI-, and PDI-based compounds (Figure 7). Similar with Figure 7a,b, as shown in Figure 7c, there are two apparent FTIR peaks corresponding to the alkyl groups of -CH3 and -CH2 (~2934 cm^−1^ and ~2848 cm^−1^) and their two-folds overtones are calculated to be ~5868 cm^−1^ and ~5696 cm^−1^ respectively, which is identical to 1704 nm and 1756 nm. As a matter of fact, in Figure 2c, the NIR reflectance has absorption peaks at around 1722 nm, which may attribute to the overtones of the alkyl group vibration. Compared with the C-H alkyl vibration groups, the C-H aromatic vibration peaks (around 3100 cm^−1^) show much lower intensity in FTIR for anhydride and amine compounds. These peaks almost disappeared in the alkyl derivatives with C8, C2C6, and C12 groups. The overtone of 3050 cm^−1^ is about 6100 cm^−1^, corresponding to 1640 nm, which contributes to the peaks around 1658 nm in Figure 3a. Although the overtone of the alkyl and aromatic groups contributes to the decreased NIR reflectance intensity to some extent around the typical peaks (1722 nm, 1658 nm etc.,) in the NIR reflectance spectra, the observation is still insufficient to explain what causes the BDI-, NDI-, PDI-based compounds with C12 and C2C8 groups to show lower NIR reflectance intensity than those of compounds with C8 groups across the entire test spectrum from 400 nm to 2500 nm, especially considering that the crystallinity degree of BDI-C12 (70%) and NDI-C12 (68%) are all higher than BDI-C8 (60%) and NDI-C8 (61%) while the particle size of BDI-C12 (42 nm) and BDI-C8 (40 nm) as well as NDI-C12 (34 nm) and NDI-C8 (31 nm) are almost identical with each other. Hence, except the main molecular backbone, some other factors including molecular packing may also affect the NIR reflectance intensity and need further investigation.

## 3. Conclusions

In this work, not only the NIR reflectance of three diimide-based compounds BDI, NDI, and PDI but also their surface morphologies, particle sizes, and molecular crystallinities are systematically investigated. Moreover, the FTIR spectra to analyze the effect of overtone on the NIR reflectance are also tested. From the above study, several conclusions could be obtained: (1) NIR reflectance intensity is mainly determined by its own conjugated backbone. For the compounds with same alkyl groups, the NIR reflectance will increase as the degree or length of the conjugated backbone increases, especially for the reflectance from 1750 nm to 2500 nm. Despite the different functional groups, NIR reflectance will vary within a certain interval for a compound with special backbone; (2) the alkyl or amine group will generally deteriorate the NIR reflectance. We believe that all BDA, NDA, and PDA exhibited relatively higher NIR reflectance than those of their derivatives with amine or alkyl groups, which is in agreement with the theory proposed by Kaur et al. [27]. The isomers with branched alkyl groups show identical or smaller NIR reflectance intensity than those of isomers with linear alkyl groups; and (3) for different compounds, crystallinity has insignificant effect on their NIR reflectance. The molecular packing or other factors may eventually play an important role in determining the NIR reflectance, however, a clear relationship is under investigation. The design of materials with the optimized or precise NIR reflectance requires a deep understanding of the structure—NIR property relationships and further study in this field is highly desirable. Moreover, compared with the other organic pigments such as azo pigments [9] and copper phthalocyanine [10], the PDI-based pigments exhibited relatively better NIR reflectance, indicating that rylene-diimide-based molecules may be potential candidates for highly effective NIR reflectance materials after appropriate structure modification.

## 4. Experimental Section

The anhydride BDA, NDA, and PDA as well as other reactants (e.g., various alkyl amine) were purchased from Sigma Aldrich or Alfa Aesar and used as received. NMR was measured on Bruker 300 MHz using CDCl_3_ or D_2_SO_4_ as solvent. SEM images were obtained by using a JEOL/JSM-6340F instrument. XRD was measured on Bruker D8 Advance XRD. The X-ray diffractometer used Cu Kα (λ = 1.5405 Angstrom) radiation with 2 θ from 10° to 70°. FTIR were taken on a FTIR Perkin Elmer Frontier. The reflectance spectra were obtained on a UV-Vis-NIR Lambda 950 instrument.

Fabrication Methodology and Reflectance Measurement: Total of 300 mg powder samples were ground homogenously with a mortar and pestle and pressed into cylinders with a diameter of 0.5 inch for the test. A 150-mm integrating sphere accessory was used to measure the NIR diffuse reflectance of the samples from 400 nm to 2500 nm.

The average reflectance was calculated according to the following equation:

Rave = Sum of Reflectance (a nm to b nm)/Range(a−b)(2)

where a and b are the starting and ending wavelengths of the range.

The grain size was calculated according to the Scherrer Equation:

D = Kλ/β cos θ(3)

where K is Scherrer constant (0.94), λ is 1.54056 Å, β is the full width at half maxima of the diffraction peak, and θ is the diffraction angle.

## Figures and Tables

**Table 1:** Average NIR reflectance of 700–1750 nm and 1750–2500 nm respectively for BDI-, NDI-, and PDI-based compounds.

Compounds	Wavelength (nm)	Anhydride (%)	NH (%)	C8 (%)	C2C6 (%)	C12 (%)
BDI-	700–1750	75	77	59	57	54
	1750–2500	23	41	25	21	20
NDI-	700–1750	76	58	77	69	68
	1750–2500	45	31	40	28	32
PDI-	700–1750	70	49	68	50	59
	1750–2500	45	29	48	31	32

**Table 2:** Particle size and crystallinity for BDI-, NDI-, and PDI-based compounds.

Compounds	Factors	Anhydride	NH	C8	C2C6	C12
BDI-	Particle Size (nm)	34	24	40	16	42
	Crystallinity (%)	42	64	60	65	70
NDI-	Particle Size (nm)	27	30	31	41	34
	Crystallinity (%)	57	71	61	71	68
PDI-	Particle Size (nm)	29	12	53	75	57
	Crystallinity (%)	43	40	64	53	62

## Supplementary Materials

The following are available online at https://www.mdpi.com/article/10.3390/ma14185269/s1, Figure S1: Reference reflectance, details of any supplementary information: ^1^ H NMR.

## References

[1] Mazhar M., Abdouss M., Zarifi F., Zargaran M. (2020). Effectiveness of perylene pigment on the reduction of energy demand of a building. Pigm. Resin. Technol..

[2] Levinson R., Berdahl P., Akbari H., Miller W., Joedicke I., Reilly J., Suzuki Y., Vondran M. (2007). Methods of creating solar-reflective nonwhite surfaces and their application to residential roofing materials. Sol. Energy Mater. Sol. Cells.

[3] Kolokotroni M., Giannitsaris I., Watkins R. (2006). The effect of the London urban heat island on building summer cooling demand and night ventilation strategies. Sol. Energy.

[4] Wong N.H., Jusuf S.K., Syafii N.I., Chen Y., Hajadi N., Sathyanarayanan H., Manickavasagam Y.V. (2011). Evaluation of the impact of the surrounding urban morphology on building energy consumption. Sol. Energy.

[5] Jose S., Joshy D., Narendranath S.B., Periyat P. (2019). Recent advances in infrared reflective inorganic pigments. Sol. Energy Mater. Sol. Cells.

[6] Wong A., Daoud W.A., Liang H.-H., Szeto Y.S. (2015). Application of rutile and anatase onto cotton fabric and their effect on the NIR reflection/surface temperature of the fabric. Sol. Energy Mater. Sol. Cells.

[7] Li Y.-Q., Mei S.-G., Byon Y.-J., Wang J.-L., Zhang G.-L. (2013). Highly solar radiation reflective Cr_2_O_3_–3TiO_2_ orange nanopigment prepared by a polymer-pyrolysis method. ACS Sustain. Chem. Eng..

[8] Sreeram K.J., Aby C.P., Nair B.U., Ramasami T. (2008). Colored cool colorants based on rare earth metal ions. Sol. Energy Mater. Sol. Cells.

[9] Yamamiya S., Horiguchi S., Ohira S., Abe Y., Zama Y., Nishikatsu H. (1987). Inventors Dainichi Color & Chem MFG Co LTD Assignee. Infrared Reflective Material. Japanese Patent.

[10] Bäbler F. (2006). Inventor Cibaspeciality, Assignee. IR Reflective Pigment Composition. U.S. Patent.

[11] Graser F. (1976). Inventor BASF AG Assignee. N-Substd. Perylenetetracarboxylic Acid Diimides—As Black Pigments for Paints, Plastics and aq. Compsns. German Patent.

[12] Erk P., Stohr A., Boehm A., Kurtz W., Mizuguchi J., Sens B. (2008). Inventors BASF Aktiengesellschaft Assignee. Black Perylene Pigments. U.S. Patent.

[13] Faulkner E.B., Schwartz R.J. (2009). High Performance Pigments.

[14] Song R.B., Wu Y., Lin Z.Q., Xie J., Tan C.H., Loo J.S.C., Cao B., Zhang J.R., Zhu J.J., Zhang Q. (2017). Living and conducting: Coating individual bacterial cells with in situ formed polypyrrole. Angew. Chem. Int. Ed. Engl..

[15] Sonmez G., Meng H., Zhang Q., Wudl F. (2003). A Highly Stable, New electrochromic polymer: Poly(1,4-bis(2-(3′,4′-ethylenedioxy) thienyl)-2-methoxy-5-2″-ethylhexyloxybenzene). Adv. Funct. Mater..

[16] Yao C.J., Zhang H.L., Zhang Q. (2019). Recent progress in thermoelectric materials based on conjugated polymers. Polymers.

[17] Zhang Q. (2020). Shooting flexible electronics. Front. Phys..

[18] Roes A.L., Alsema E.A., Blok K., Patel M.K. (2009). Ex-ante environmental and economic evaluation of polymer photovoltaics. Prog Photovolt.

[19] Brass D.M., Palmer S.M. (2017). Models of toxicity of diacetyl and alternative diones. Toxicology.

[20] Chen W., Song Y., Zhang L., Liu M., Hu X., Zhang Q. (2018). Thiophene-fused-heteroaromatic diones as promising NIR reflectors for radiative cooling. Angew. Chem. Int. Ed. Engl..

[21] Carlotti M., Gullo G., Battisti A., Martini F., Borsacchi S., Geppi M., Ruggeri G., Pucci A. (2015). Thermochromic polyethylene films doped with perylene chromophores: Experimental evidence and methods for characterization of their phase behaviour. Polym. Chem..

[22] Donati F., Pucci A., Cappelli C., Mennucci B., Ruggeri G. (2008). Modulation of the optical response of polyethylene films containing luminescent perylene chromophores. J. Phys. Chem. B.

[23] Wurthner F. (2004). Perylene bisimide dyes as versatile building blocks for functional supramolecular architectures. Chem. Commun..

[24] Raj M.R., Margabandu R., Mangalaraja R.V., Anandan S. (2017). Influence of imide-substituents on the H-type aggregates of perylene diimides bearing cetyloxy side-chains at bay positions. Soft Matter.

[25] Kaur B., Quazi N., Ivanov I., Bhattacharya S.N. (2012). Near-infrared reflective properties of perylene derivatives. Dyes Pigm..

[26] Mazhar M., Abdouss M., Gharanjig K., Teimuri-Mofrad R. (2016). Synthesis, characterization and near infra-red properties of perylenebisimide derivatives. Prog. Org. Coat..

[27] Kaur B., Bhattacharya S.N., Henry D.J. (2013). Interpreting the near-infrared reflectance of a series of perylene pigments. Dyes Pigm..

[28] Mazhar M., Abdouss M., Gharanjig K., Teimuri-Mofrad R., Zargaran M. (2016). Effects of isomerism on near infrared properties of perylene bisimide derivatives. J. Coat. Technol Res..

[29] Mahmoudi Meymand F., Mazhar M., Abdouss M. (2018). Investigation of substituent effect on cool activity of perylene bisimide pigments. J. Coat. Technol Res..

[30] Martini F., Geppi M., Barcaro G., Monti S., Contiero L., Ruggeri G., Lessi M., Pucci A., Bellina F., Borsacchi S. (2020). Structure and dynamics of perylene bisimide pigments for “cool” organic coatings by solid-state NMR: A combined experimental and DFT study. J. Phys. Chem. C.

[31] Martini F., Minei P., Lessi M., Contiero L., Borsacchi S., Ruggeri G., Geppi M., Bellina F., Pucci A. (2020). Structural order and NIR reflective properties of perylene bisimide pigments: Experimental evidences from a combined multi-technique study. Dyes Pigm..

[32] Minei P., Lessi M., Contiero L., Borsacchi S., Martini F., Ruggeri G., Geppi M., Bellina F., Pucci A. (2020). Boosting the NIR reflective properties of perylene organic coatings with thermoplastic hollow microspheres: Optical and structural properties by a multi-technique approach. Sol. Energy.

[33] Song Y., Chen W., Lim X.M., Hu X., Liu M., Zhang Q. (2019). Electronic configuration in outset orbitals of doping elements plays as a key factor in tuning near infrared reflection of YMn_0.9_M_0.1_O_3_ (M = Cr, Mn, Fe, Co, Al, Ga and In). J. Solid State Chem..

[34] Seckin T., Özdemir I., Köytepe S., Gürbüz N. (2009). Preparation and Catalytic Properties of a Ru(II) Coordinated Polyimide Supported by a Ligand Containing Terpyridine Units. J. Inorg. Organomet. Polym. Mater..

[35] Yang T.F., Huang S.H., Chiu Y.P., Chen B.H., Shih Y.W., Chang Y.C., Yao J.Y., Lee Y.J., Kuo M.Y. (2015). Pyromellitic dithioimides: Thionation improves air-stability and electron mobility of N-type organic field-effect transistors. Chem. Commun..

[36] Shi Y., Tang H., Jiang S., Kayser L.V., Li M., Liu F., Ji F., Lipomi D.J., Ong S.P., Chen Z. (2018). Understanding the electrochemical properties of naphthalene diimide: Implication for stable and high-rate lithium-ion battery electrodes. Chem. Mater..

[37] Jung B.J., Lee K., Sun J., Andreou A.G., Katz H.E. (2010). Air-operable, high-mobility organic transistors with semifluorinated side chains and unsubstituted naphthalenetetracarboxylic diimide cores: High mobility and environmental and bias stress stability from the perfluorooctylpropyl side chain. Adv. Funct. Mater..

[38] Chen W., Zhang J., Long G., Liu Y., Zhang Q. (2015). From non-detectable to decent: Replacement of oxygen with sulfur in naphthalene diimide boosts electron transport in organic thin-film transistors (OTFT). J. Mater. Chem. C.

[39] Chen W., Yang X., Long G., Wan X., Chen Y., Zhang Q. (2015). A perylene diimide (PDI)-based small molecule with tetrahedral configuration as a non-fullerene acceptor for organic solar cells. J. Mater. Chem. C.

[40] Black D.B., Lovering E.G. (1977). Estimation of the degree of crystallinity in digoxin by X-ray and infrared methods. J. Pharm. Pharmacol..

[41] Shah B., Kakumanu V.K., Bansal A.K. (2006). Analytical techniques for quantification of amorphous/crystalline phases in pharmaceutical solids. J. Pharmacol. Sci..

