# Supplementary material for: Insights into the Intrinsic Factors Affecting the NIR Reflectance Based on Rylene Diimide Molecules
Source: Materials (Basel). 2021 Sep 13;14(18):5269. doi: 10.3390/ma14185269 (PMC8466077; doi:10.3390/ma14185269)
Supplement: Supplementary file 1 [file materials-14-05269-s001.zip › materials-1336825-supplementary.pdf]

materials

Supplementary Information

Insights into the Intrinsic Factors Affecting the NIR Reflectance Based on Rylene Diimide Molecules

Weili Zeng [1,†], Yujie Song [2,†], Jianning Zhang [2], Hong Chen [1], Ming Liu [3,*] and Wangqiao Chen [1,*]

[1] Guangdong Provincial Key Laboratory of Optical Information Materials and Technology & National Center for International Research on Green Optoelectronics, South China Academy of Advanced Optoelectronics, South China Normal University, Guangzhou 510006, China; 2019023280@m.scnu.edu.cn (W.Z.); 2020023689@m.scnu.edu.cn (H.C.)
[2] Engineering Laboratory of Advanced Energy Materials, Ningbo Institute of Materials Technology & Engineering, Chinese Academy of Sciences, Ningbo 315201, China; songyujie@nimte.ac.cn (Y.S.); zhangjianning@nimte.ac.cn (J.Z.)
[3] School of Chemistry and Chemical Engineering, Harbin Institute of Technology, Heilongjiang 150001, China
* Correspondence: wqchen@m.scnu.edu.cn (W.C.); liuming0117@hit.edu.cn (M.L.)
† These authors contributed equally to this work.

Citation: Chen, W.; Zeng, W.; Zhang, J.; Chen, H.; Song, Y.; Liu, M.; Zhou, G.; Zhang, Q. Insights into the Intrinsic Factors Affecting the NIR Reflectance Based on Rylene Diimide Molecules . *Materials* **2021**, *14*, 5269. https://doi.org/10.3390/ma14185269

Academic Editor: Werner Blau

Received: 27 July 2021
Accepted: 10 September 2021
Published: 13 September 2021

Publisher's Note: MDPI stays neutral with regard to jurisdictional claims in published maps and institutional affiliations.

Copyright: © 2021 by the authors. Submitted for possible open access publication under the terms and conditions of the Creative Commons Attribution (CC BY) license (https://creativecommons.org/licenses/by/4.0/).

General Information

The anhydride BDA, NDA and PDA as well as other reactants (e. g. various alkyl amine) were purchased from Sigma Aldrich or Alfa Aesar and used as received. NMR was measured on Bruker 300MHz using $CDCl_3$ or D_2SO_4 as solvent. SEM images were obtained by using a JEOL/JSM-6340F instrument. XRD was measured on Bruker D8 Advance XRD. The X-ray Diffractometer used Cu Kα (λ = 1.5405 Angstrom) radiation with 2θ from 10° to 70°. FTIR were taken on a FTIR Perkin Elmer Frontier. The reflectance spectra were obtained on a UV-Vis-NIR Lambda 950 instrument. A 150 mm integrating sphere accessory was used to measure the diffuse reflectance of the samples. 300 mg powder samples were grinded with a mortar and pestle homogenously and pressed into cylinders with a diameter of 0.5 inch for the test.

The average reflectance was calculated according to the following equation:

$$R_{ave} = \text{Sum of Reflectance (a nm to b nm)/Range(a-b)}$$

Where a and b are the starting and end wavelength of the range.

The grain size was calculated according to the Scherrer Equation:

$$D = K\lambda/\beta \cos\theta$$

where K is Scherrer constant (0.94), λ is 1.54056 Å, β is the full width at half maxima of the diffraction peak, and θ is diffraction angle.

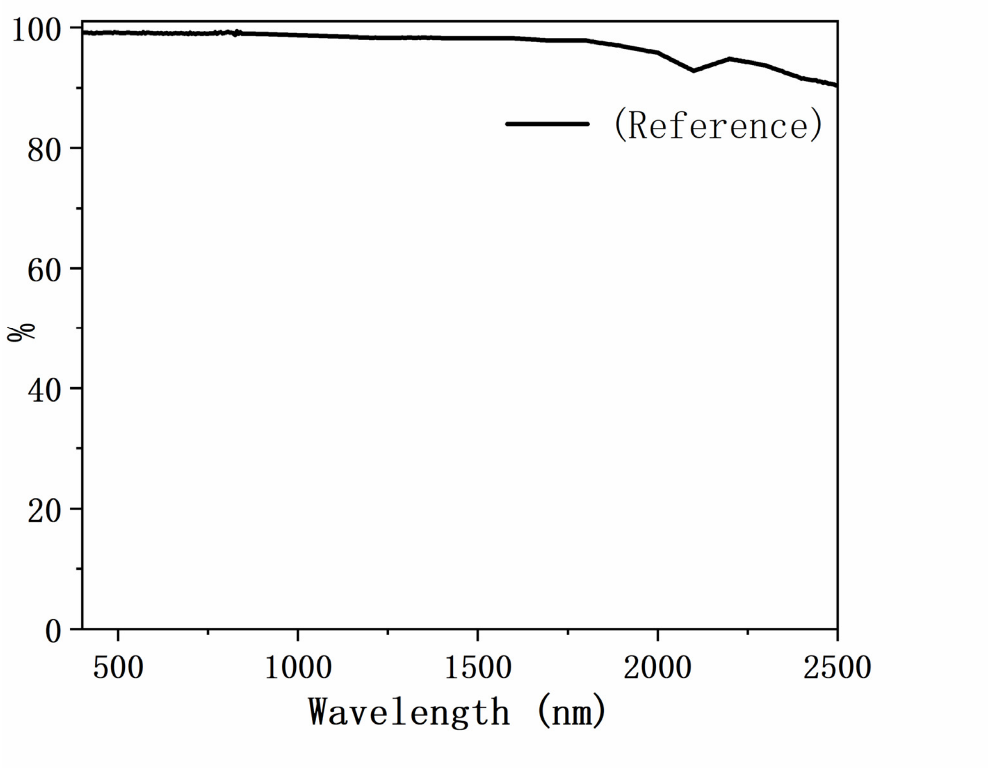

Figure S1. Reference reflectance

^{1}H NMR Spectra

BDI-C_8

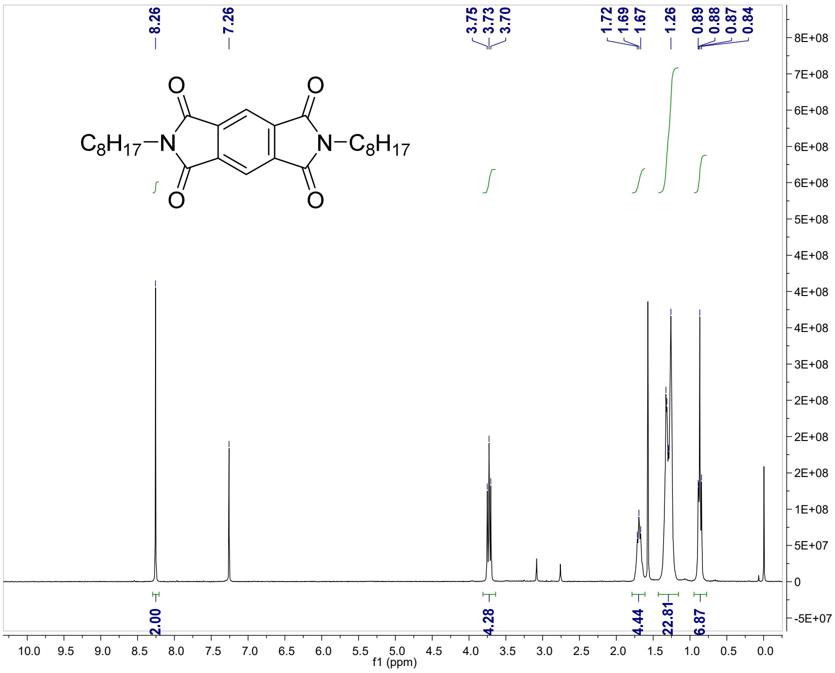

^{1}H NMR (300 MHz, $CDCl_3$): δ = 8.26 (s, 2H), 3.73 (t, 4H), 1.72-1.67 (m, 4H), 1.32-1.26 (m, 20H), 0.89-0.84 (m, 6H)

BDI-C_2C_6

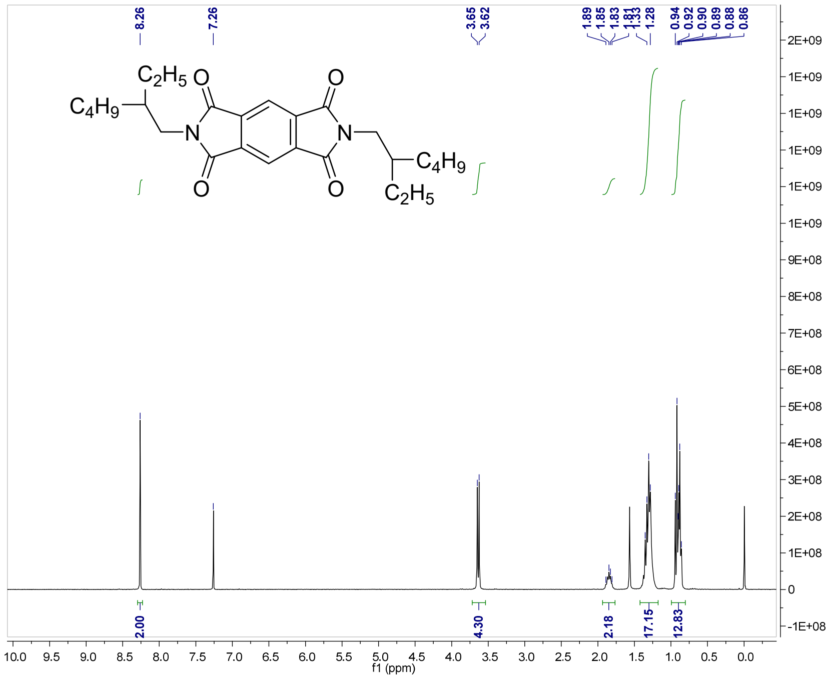

^{1}H NMR (300 MHz, $CDCl_3$): δ = 8.26 (s, 2H), 3.33 (d, 4H), 1.81-1.89 (m, 2H), 1.35-1.28 (m, 16H), 0.94-0.86 (m, 12H)

BDI-C_{12}

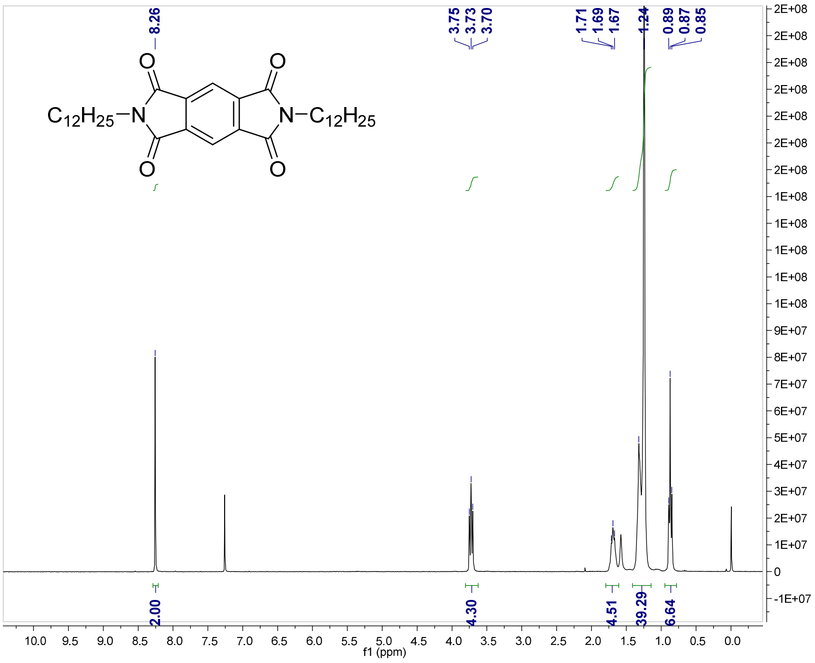

^1H NMR (300 MHz, $CDCl_3$): δ = 8.26 (s, 2H), 3.73 (d, 4H), 1.71-1.67 (m, 4H), 1.32-1.24 (m, 36H), 0.89-0.85 (m, 6H)

NDI-C_8

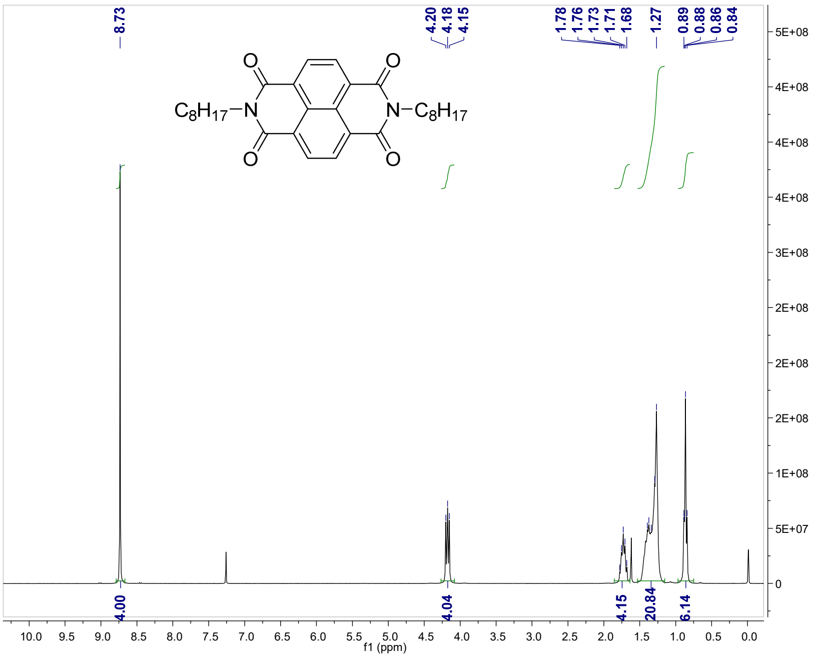

^1H NMR (300 MHz, $CDCl_3$): δ = 8.73 (s, 4H), 4.18 (t, 4H), 1.78-1.68 (m, 4H), 1.39-1.27 (m, 20H), 0.89-0.84 (m, 6H)

NDI-C_2C_6

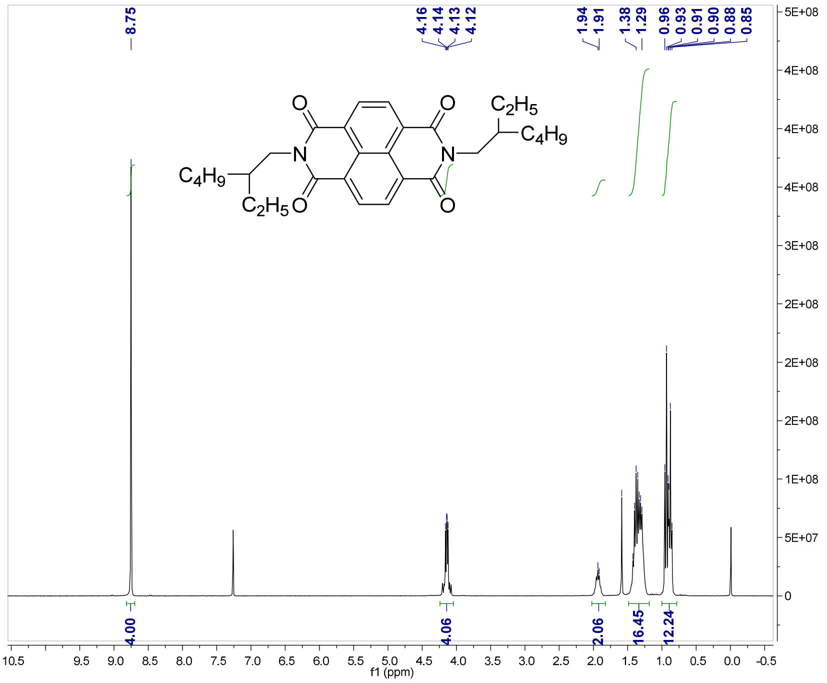

^{1}H NMR (300 MHz, $CDCl_3$): δ = 8.75 (s, 4H), 4.16-4.12 (d, 2H), 1.89-1.98 (m, 2H), 1.40-1.29 (m, 16H), 0.96-0.85 (m, 12H)

NDI-C_{12}

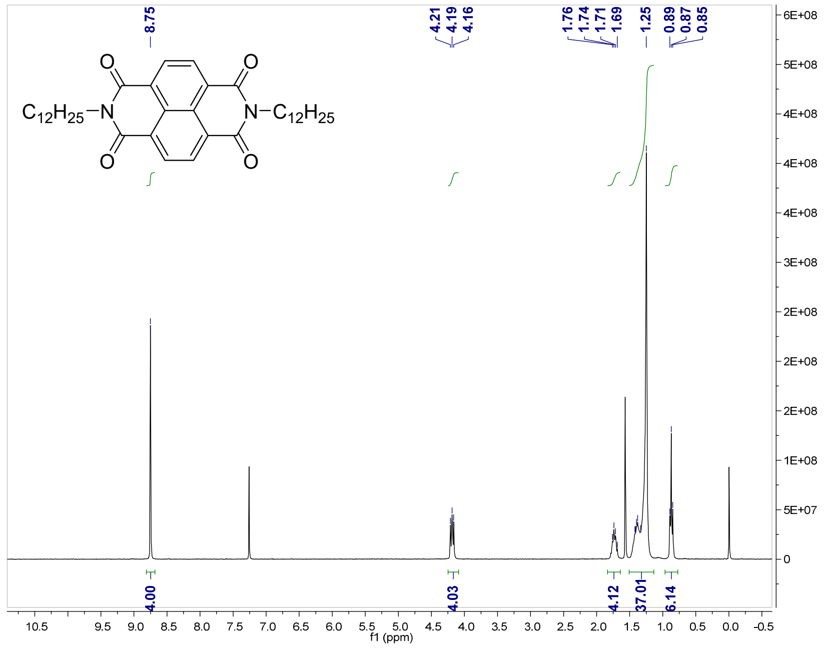

^{1}H NMR (300 MHz, $CDCl_3$): δ = 8.75 (s, 4H), 4.19 (t, 4H), 1.76-1.69 (m, 4H), 1.42-1.25 (m, 36H), 0.89-0.85 (m, 6H)

PDI-C8

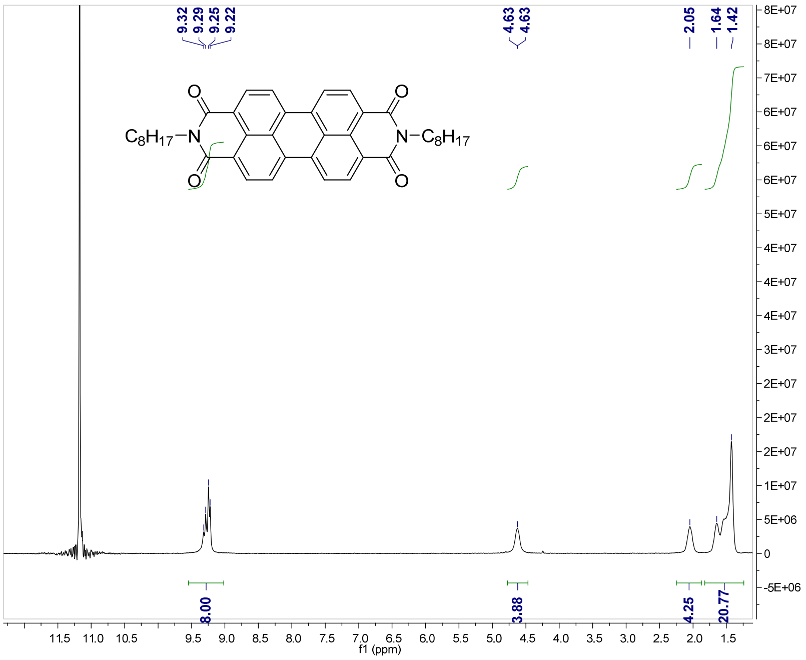

^{1}H NMR (300 MHz, D_2SO_4): δ = 9.32-9.22 (m, 8H), 4.63 (s, 4H), 2.05 (s, 4H), 1.64-1.42 (m, 20H), 0.89-0.84 (m, 6H)

PDI-C2C6

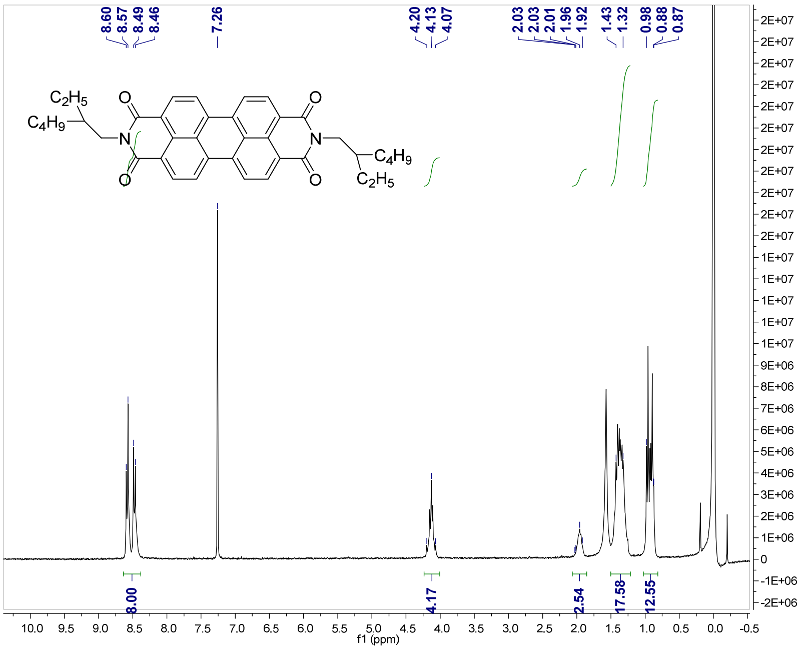

^{1}H NMR (300 MHz, $CDCl_3$): δ =δ = 8.60-8.46 (m, 8H), 4.20-4.07 (d, 2H), 2.03-1.92 (m, 2H), 1.43-1.32 (m, 16H), 0.98-0.87 (m, 12H)

PDI-C_{12}

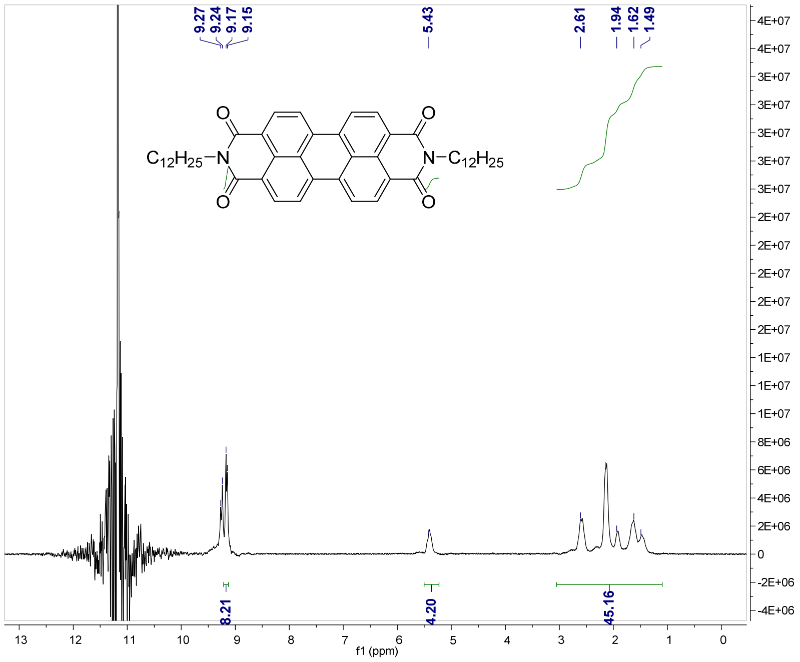

^{1}H NMR (300 MHz, D_2SO_4): δ = 9.27-9.15 (m, 8H), 5.43 (s, 4H), 2.61-1.49 (m, 46H)
